# Zinc Phosphate Microparticles against Nosocomial and Oral Bacteria: Synthesis, Analytical Characterization, and Biocompatibility

**DOI:** 10.1021/acsomega.5c02071

**Published:** 2025-06-12

**Authors:** Lorena Reyes-Carmona, Margherita Izzi, Rosaria Anna Picca, Maria Chiara Sportelli, Gina Prado-Prone, Phaedra Silva-Bermudez, Sandra E. Rodil, Nicola Cioffi, Argelia Almaguer-Flores

**Affiliations:** † Laboratorio de Biointerfases, División de Estudios de Posgrado e Investigación, Facultad de Odontología, Universidad Nacional Autónoma de México, Circuito exterior s/n, Ciudad Universitaria, CDMX 04510, México; ‡ Dipartimento di Chimica, Università degli Studi di Bari Aldo Moro, Via E. Orabona 4, Bari 70125, Italia; § Unidad de Ingeniería de Tejidos, Terapia Celular y Medicina Regenerativa, Instituto Nacional de Rehabilitación Luis Guillermo Ibarra Ibarra, Av. México-Xochimilco No. 289 Col. Arenal de Guadalupe, CDMX C.P. 14389, México; ∥ Instituto de Investigaciones en Materiales, Universidad Nacional Autónoma de México, Circuito exterior s/n, Ciudad Universitaria, CDMX 04510, México

## Abstract

Nosocomial bacteria represent a significant global health problem. In addition, oral pathogens causing oral infections, such as periodontitis and peri-implantitis, are the main cause of the failure of oral implant treatments, mainly due to bacterial resistance related to the indiscriminate use of antibiotics in recent years. Therefore, identifying antimicrobial and biocompatible agents, such as some zinc-derived compounds, represents a promising alternative to the development of new antibacterial biomaterials. In this study, zinc phosphate microparticles were synthesized by chemical precipitation and characterized by FTIR, DLS, TEM, XRD, and XPS. Their antibacterial effect was evaluated against nosocomial and oral bacteria, while their biocompatibility was assessed using human fibroblasts and osteoblasts. The results showed zinc phosphate microparticles with elongated morphologies, a hopeite crystal structure with an average crystallite size of about 35 nm, a hydrodynamic diameter of approximately 4.8 μm, and a ζ-potential close to neutrality. Regarding the antibacterial properties, zinc phosphate microparticles showed high antibacterial activity against the eight different bacterial species evaluated. In almost all species, an inhibition percentage close to 100% was observed, depending on the concentration, while in the biocompatibility tests, particle concentrations between 0.05 and 0.4 mg/mL were not cytotoxic to either of the eukaryotic cell types evaluated. These findings suggest that zinc phosphate microparticles synthesized by chemical precipitation possess antibacterial properties against pathogens associated with nosocomial and oral infections and exhibit biocompatibility with human fibroblasts and osteoblasts. Therefore, zinc phosphate microparticles have the potential for diverse applications in the medical and dental fields due to their antibacterial properties and biocompatibility.

## Graphical abstract

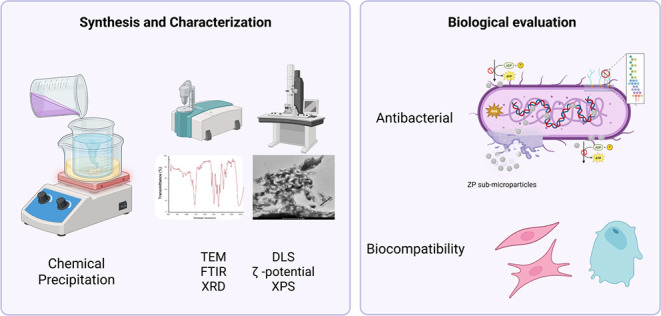

## 1 Introduction

Nosocomial or hospital-acquired infections (HAIs) are transmitted while receiving or providing medical attention in healthcare centers, such as hospitals and clinics. They are a significant global health problem due to the high morbidity and mortality rates, and the economic and social implications.^1,2^ The main etiology of HAIs is associated with bacterial infections, which are responsible for almost 90% of HAI cases in healthcare environments. Among the most common pathogens are aerobic pathogens Escherichia coli (E. coli), Pseudomonas aeruginosa (P. aeruginosa), and Staphylococcus aureus (S. aureus), with E. coli being the most frequent strain in urinary infections and S. aureus in wound and blood infections.^3–5^

In the same way, oral infections such as periodontitis and peri-implantitis are characterized by dysbiotic biofilms present in the oral cavity.^6,7^ Periodontal infections represent one of the most prevalent oral diseases globally, with a microbial etiology affecting an estimated 20% to 50% of the world’s population.^8,9^ Within this context, the primary etiology of peri-implantitis is related to the bacterial colonization of dental implants. This colonization triggers inflammatory responses that can disrupt, or ultimately lead to the loss of, osseointegration, a biological failure frequently resulting in unsuccessful dental rehabilitation that limits clinical success.^10^ The key pathogenic species most commonly associated with both periodontitis and peri-implantitis include Aggregatibacter actinomycetemcomitans (A. actinomycetemcomitans) and Porphyromonas gingivalis (P. gingivalis). Moreover, the incidence of peri-implantitis is increased in individuals with poor oral hygiene habits.^11,12^

While most nosocomial infections and periodontal treatments are effectively managed using antimicrobial agents such as antibiotics and antiseptics, the excessive and indiscriminate use of broad-spectrum antibiotics has increased bacterial resistance in recent years.^13^ Furthermore, the bacterial profile of hospital-acquired infections has evolved over time, exhibiting increasingly dynamic and complex resistance patterns. This evolution is marked by the emergence and spread of multidrug-resistant organisms (MDROs), including species from the *Staphylococcus* and *Pseudomonas* species with intrinsic resistance mechanisms and various strains of *Enterobacteriaceae* reflecting constantly changing resistance patterns and new clinical challenges.^14^

Consequently, a multitude of antimicrobial strategies have been explored to address the growing challenges posed by both nosocomial infections and antibiotic resistance. These alternative approaches primarily focus on metal and metal oxide nanomaterials, including nanoparticles^15,16^ and nanocoatings,^17,18^ and polymeric materials,^19^ bacteriophages,^20^ photocatalysis,^21^ and antimicrobial peptides.^22–24^ In addition, novel antimicrobial agents, including zinc-derived compounds, have gained importance as an alternative to traditional therapeutic methods due to their antibacterial, antifungal, and antiviral properties.^25^

Zinc (Zn) is an element that has been extensively investigated in biomedical research; due to its rapid oxidation under ambient conditions, zinc is commonly incorporated into biomaterials in the form of zinc oxide (ZnO). Its biomedical relevance is largely attributed to its broad antimicrobial properties, making it valuable in both topical and systemic treatments.^26^ In addition, ZnO in nano- or microscale exhibits antibacterial effects against several bacterial pathogens.^27–30^ This antibacterial property is associated with the fact that zinc-derived materials have the capacity to release Zn^2+^ ions to generate reactive oxygen species (ROS), and electrostatic interactions between positively charged ZnO materials and negatively charged bacterial cell walls all contribute to its bactericidal action. Furthermore, the internalization of ultrasmall nanoparticles may lead to cell wall disruption and metabolic imbalance, ultimately causing cell death.^31–33^

Another zinc-derived material of interest is zinc phosphate (ZP), which includes mineral forms such as hopeite, parahopeite, and tarbuttite.^34^ ZP possesses several valuable properties including anticorrosive^35^ and electrocatalytic properties^35,36^ and the ability to enhance the strength and durability of materials like wastewater pumps.^37^ In the biomedical field, ZP has also demonstrated potential as a nanocarrier for the delivery of traditional chemotherapeutic agents like oxaliplatin (OXPN), contributing to improved drug stability.^38^ Beyond its mechanical and pharmacological applications, ZP has shown promising biological properties, particularly its antibacterial activity^39^ and biocompatibility properties.^40^

Recently, ZP in the form of micro- and nanoparticles (NPs) has been developed, and its antibacterial potential has been evaluated against various bacterial species such as Staphylococcus aureus (S. aureus), Escherichia coli (E. coli), Pseudomonas aeruginosa (P. aeruginosa), Methicillin-resistant Staphylococcus aureus (MRSA).^41–43^ ZP NPs have been shown to reduce the virulence factors of the E. coli strain present in piglets, preventing farm contamination.^44^ Despite the favorable antibacterial results achieved with zinc phosphate in micro- or nanoparticle forms, there is a limited number of studies evaluating their effectiveness against oral pathogens, and cytotoxicity.^39,45^ Therefore, this study aims to further investigate the bactericidal capacity of ZP microparticles against oral pathogens associated with two main etiologies of oral infections: periodontitis and dental caries. Additionally, the biocompatibility properties of ZP were evaluated by using human dermal fibroblasts and osteoblasts.

Various synthesis methods have been extensively explored for the fabrication of nano- and micro-zinc phosphate (ZP) materials. Among them, the dipping method utilizing phosphating baths,^46^ sonochemical methods,^47^ chemical precipitation at room temperature or with thermal assistance.^48,49^ Additionally, biological synthesis methods have gained attention for their environmentally friendly and sustainable nature,^50^ among others. Each of these methodologies offers specific advantages in controlling the size, morphology, and other characteristics of the particles.

It is important to highlight that the majority of the seminal works on zinc phosphate have predominantly focused on its application as a corrosion-resistant coating, aiming to enhance the durability and protection of metal substrates via nanoscale ZP coatings.^46,51,52^ In this study, chemical precipitation was chosen as the preferred synthesis route due to its operational simplicity, cost-effectiveness, and potential scalability. Despite the antimicrobial properties of ZP, for developing safe antibacterial treatments, further investigation is needed into its antimicrobial capacities against a broader range of bacterial pathogens, and biocompatibility toward eukaryotic cells.

## 2 Experimental Section

### 2.1 Zinc Phosphate (ZP) Microparticles Synthesis

The zinc phosphate (ZP) microparticles were synthesized by the chemical precipitation method following the reference by Horky et al.^42^ with some modifications. Briefly, 4.46 g (0.5 M) of Zn­(NO_3_)_2_·6H_2_O (Sigma-Aldrich, reagent grade 98%) was dissolved in 50 mL of Milli-Q water, and the solution was heated to 60 °C in an oil bath. Then, Na_2_HPO_4_ (0.5 M; anhydrous, Sigma-Aldrich, reagent grade plus ≤99.99%) was dissolved in 20 mL of Milli-Q water and sonicated for 30 min. Next, the solution containing the phosphate precursor was added to the Zn­(NO_3_)_2_·6H_2_O solution while being stirred, and a white precipitate formed immediately. The suspension was stirred for 2 h; after stirring, Milli-Q water was added to reach 100 mL. The pH of the solution was measured at the beginning (pH = 3.8) and at the end (pH = 2.7) of the synthesis. Later, 10 mL of the sample was recovered, centrifuged for 20 min, washed with Milli-Q water, and centrifuged again. Finally, the sample was dried overnight in an oven at 120 °C, and a white powder was obtained (Figure 1).

**Figure 1:**
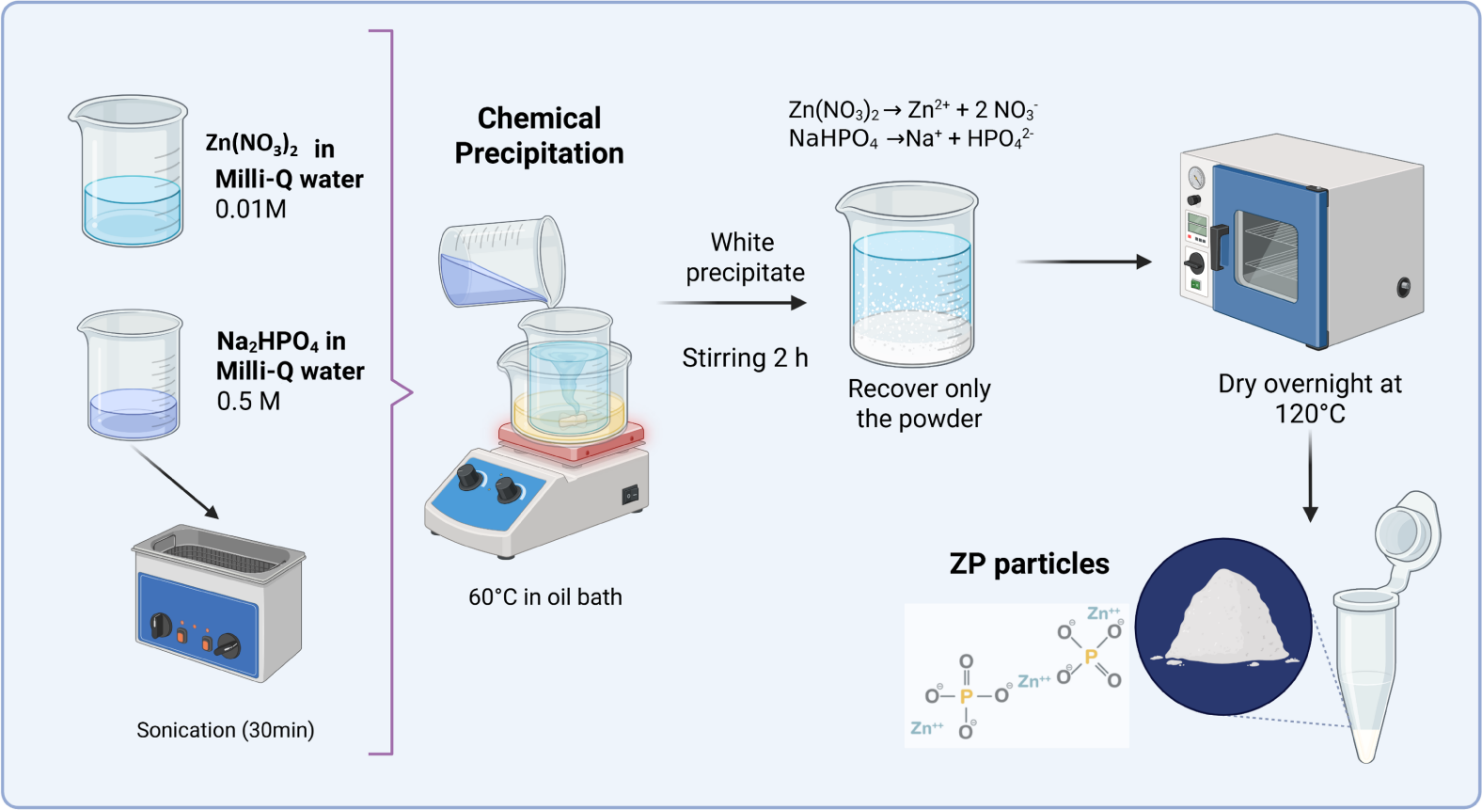
Representative scheme of the synthesis process of zinc phosphate (ZP) microparticles via the wet chemical precipitation method. The precursor reagents used in the synthesis are shown on the left side, while the scheme on the right side details the key steps of the procedure: the mixing of solutions, the formation of the characteristic white precipitate, and the stirring and drying times required before collection and storage of the final product in powder form (ZP particles). Created in BioRender. Reyes-Carmona, L. (2025) https://biorender.com/uk2af7b.

### 2.2 Analytical Characterization

The chemical composition of the resultant powder was analyzed by attenuated total reflectance infrared spectroscopy (ATR-FTIR) using a PerkinElmer Spectrum Two spectrometer (Milan, Italy). A diamond prism with one reflection was used as the internal reflection element. The measurements were taken at a resolution of 2 cm^–1^, scanning from 4000 to 400 cm^–1^ and acquiring 32 scans. Background correction was performed against air.

Hydrodynamic diameter and ζ-potential of microparticles in suspension were measured using a Zetasizer Nano ZS from Malvern Instruments (Rome, Italy). Aqueous suspensions of ZP particles were suspended in Milli-Q water at a concentration of 0.5 g/L. The sample holder was kept at a constant temperature of 25 °C using a Peltier device. Laser Doppler electrophoresis (LDE) was conducted with forward scattering at an angle of 17°, and measurements were taken using a capillary cell, following the methodology reported by Sportelli et al.^53^

The morphology and average size of particles were identified by using a transmission electron microscopy (TEM) instrument (FEI Tecnai 12, Hillsboro, OR, USA) (high voltage: 120 kV; filament: LaB_6_).

The crystalline structure was analyzed by X-ray diffraction (XRD) using a Bruker D8 XRD diffractometer with CuKα radiation (λ = 0.15418 nm) in a 2θ range from 5° to 60°. The average crystallite size (*D*) was calculated from the three main diffraction peaks, corresponding to the (020), (040), and (311) planes, using Scherrer′s’ equation:

1 $$D=\frac{K\lambda }{\beta \cos\theta }$$

where *K* is the Scherrer constant (0.94), λ is the wavelength of the X-ray radiation, β is the full width at half maximum (fwhm), and θ is the Bragg angle.

X-ray photoelectron spectroscopy (XPS) analysis was performed with a PHI Versaprobe II instrument (monochromatic Al Kα source 1486.6 eV, 50 W, 200 μm spot). The pass energy was set at 46.95 eV for acquiring high-resolution (HR) spectra, binding energy (BE) referred to the aliphatic component of C 1s at 284.8 eV, and quantification and peak fitting were carried out with Multipak v.9.9.3 software.^54^

### 2.3 Antibacterial Assay

#### 2.3.1 Bacteria Strains

Antibacterial tests were performed using eight bacterial strains (four aerobic nosocomial and four anaerobic oral pathogens) from the American Type Cell Culture Collection (ATCC). Nosocomial bacteria evaluated were Escherichia coli (E. coli) ATCC 33780, Pseudomonas aeruginosa (P. aeruginosa) ATCC 43636, Staphylococcus aureus (S. aureus) ATCC 25923, and Staphylococcus epidermidis (S. epidermidis) ATCC 14990. Each strain was cultured individually on trypticase soy agar (TSA) (BBL, Becton Dickinson) plates and incubated for 24 h at 37 °C under aerobic conditions.

Anaerobic oral bacteria tested were Actinomyces israelii (A. israelii) ATCC 12102, Aggregatibacter actinomycetemcomitans serotype b (*A. a. b*) ATCC 43718, Porphyromonas gingivalis (P. gingivalis) ATCC 33277, and Streptococcus mutans *(* S. mutans) ATCC 25175. The strains were individually cultured on enriched agar plates with *Mycoplasma* agar (Sigma-Aldrich) supplemented with 5 μg/mL hemin (Sigma-Aldrich), 0.3 μg/mL menadione (Sigma-Aldrich), and 5% defibrinated lamb blood (Microlab). The bacterial cultures were incubated for 7 days at 35 °C under anaerobic conditions (80% N_2_, 10% CO_2_, and 10% H_2_). The optical density (OD) was adjusted to 1 at λ = 600 nm in a spectrophotometer (BioPhotometer D30, Eppendorf).

#### 2.3.2 Antibacterial Assay

Bacterial suspensions (1 × 10^5^ cells/mL) obtained from pure cultures of each bacterial strain tested were seeded with different concentrations of ZP microparticles (0.05, 0.1, 0.2, 1, 1.5, and 3 mg/mL) (previously suspended in sterile water) and incubated with the appropriate culture broth media at 35 °C under agitation for 24 h (under aerobic or anaerobic conditions depending on the strain). Afterward, four-serial dilutions were performed, and 5 μL of each concentration was seeded on agar plates and then incubated for 24 h (aerobic bacteria) or 7 days (anaerobic bacteria) depending on the species. Culture broth media with 0.2% chlorhexidine was used as positive control, while as negative control, the strains were cultured only with culture broth media without ZP microparticles. After incubation, the number of colony forming units (CFUs) was visually quantified, and the logarithmic reduction (log reduction) and the percentage reduction were calculated with the following equations:

2 $$\mathrm{number of CFUs}/\mathrm{mL}=\frac{\#\mathrm{CFUs}}{V}\times \left(\mathrm{IDF}\right)$$

where CFUs is the number of colony forming units, *V* is the volume seeded on the agar plates (in all cases 0.005 mL), and IDF is the inverse dilution factor (corresponds to the inverse number of the dilution at which it was possible to quantify the CFUs).

3 $$\log\;\mathrm{reduction}=\log\;10=\left(\frac{A}{B}\right)$$

where *A* is the number of CFUs/mL in the negative control (culture broth media with bacteria and no ZP microparticles) and *B* is the number of CFUs/mL that grew on interaction with ZP microparticles.

4 $$\mathrm{Inhibition percentage}=\frac{\left(A-B\right)\times 100}{A}$$

where *A* is the number of CFUs that grew in the negative control (culture broth media with bacteria and no ZP microparticles) and *B* is the number of CFUs that grew on interaction with ZP microparticles.

### 2.4 Cytotoxicity Assays

The cytotoxic potential of ZP microparticles was evaluated by measuring the viability of human dermal fibroblasts (HDFa; ATCC PCS-201-012) and human osteoblasts (hFOB; ATCC CRL-3602) after exposure to different concentrations of ZP microparticle suspensions using the colorimetric MTT (3-(4,5-dimethylthiazolyl-2)-2,5-diphenyltetrazolium bromide) assay (MTT) (Sigma-Aldrich) based on the ISO 10993-5 guidelines.

Cell cultures at ≈80% confluency were treated with 0.05% trypsin −0.02% EDTA for primary cells (ATCC PCS-999-003) for HDFa, and 0.25% trypsin-EDTA (Gibco) for hFOB, and collected by centrifugation. Cells were seeded in 96-well tissue culture plates, at a density of 1 × 10^4^ cells per well, with Dulbecco’s Modified Eagle’s Medium F12 (DMEM-F12; Gibco) supplemented with 10% v/v Fetal Bovine Serum (FBS; Gibco) and 1% v/v penicillin–streptomycin (Gibco) or 3% v/v Geneticin G418 (ATCC) for HDFa or hFOB, respectively. Then, cell cultures were incubated for 24 h at 37 °C in a 5% CO_2_ atmosphere.

After the incubation period, the culture media was removed and replaced with ZP microparticle suspensions at different concentrations: 0, 0.05, 0.1, 0.2, 0.4, 1, 1.5, and 3 mg/mL (microparticles suspended in culture media). Cells cultured with fresh culture media without ZP microparticles were used as positive control samples (ctrl; no cytotoxicity), and the two highest concentrations were previously filtered to minimize the extra absorbance produced from the largest particles in the suspensions. Cells with or without the ZP microparticle suspensions were incubated again for 24 h at 37 °C and 5% CO_2_. Following this incubation, the supernatants were discarded and replaced with an MTT: culture media solution (1:10) was incubated for 3 h. Then, formazan crystals produced by metabolically active cells were solubilized in 100 μL of an isopropyl alcohol (ISO; Sigma-Aldrich, Bioreagent, ≤99.5%) and dimethyl sulfoxide (DMSO; Sigma-Aldrich, ReagentPlus, ≤99.5%) solution (1:1), and the optical density (OD) at λ = 570 nm was measured using a microplate spectrophotometer (Synergy HTX BioTek). To calculate cell viability (%), the following equation was used:

5 $$\mathrm{Cell viability}\;\left(\%\right)=\left(\frac{{\mathrm{OD}}_{\exp}}{{\mathrm{OD}}_{\mathrm{ctrl}}}\right)\times 100$$

where OD_exp_ = optical density of the solubilized formazan produced by cells exposed to the different concentrations of ZP microparticles suspended in culture medium and OD_ctrl_ = optical density of the solubilized formazan produced by cells cultured with culture medium with no ZP microparticles.

### 2.5 Statistical Analysis

The biological experiments were performed in triplicate and repeated at least twice. The results were expressed as the mean ± standard error of the mean, and statistically significant differences between the experimental groups compared to the control group were determined by one-way analysis of variance (ANOVA) and Dunnett’s post hoc test for multiple comparisons, using a 5% significance level (*p* < 0.05) using the GraphPad Prism 5.1 software.

## 3 Results and Discussion

### 3.1 Analytical Characterization of Zinc Phosphate (ZP) Microparticles

Figure 2 shows the FTIR spectrum of the ZP microparticles synthesized by chemical precipitation and the FTIR spectra of the precursor reagents used in the synthesis: disodium hydrogen phosphate and zinc nitrate hexahydrate.

**Figure 2:**
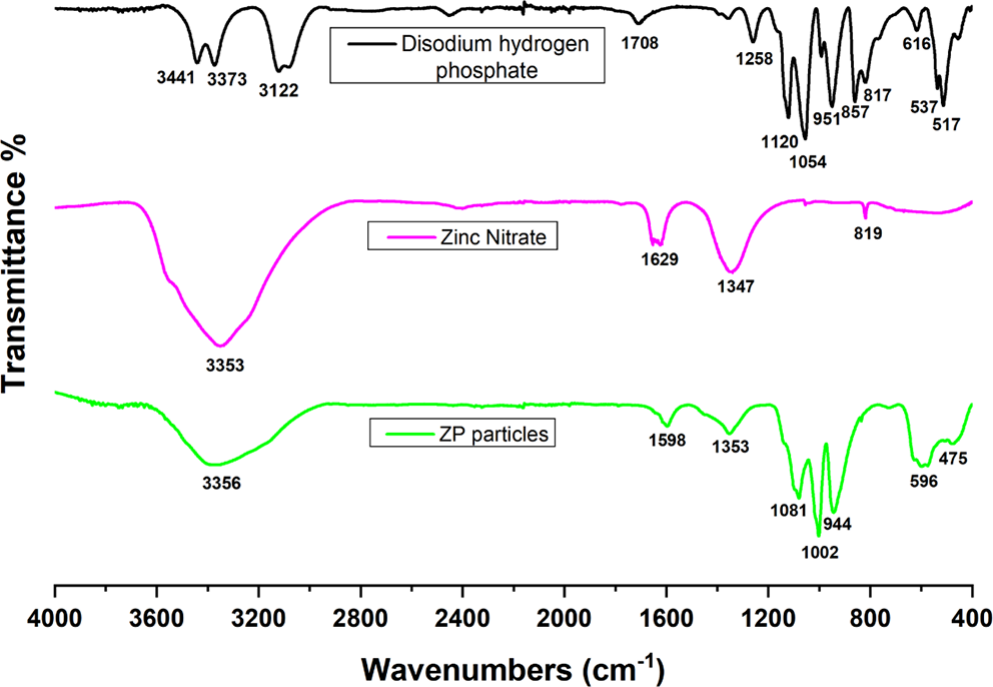
Spectra of zinc phosphate microparticles obtained by chemical precipitation synthesis and of their precursors. The upper spectrum (black) corresponds to the disodium hydrogen phosphate precursor, the middle spectrum (pink) corresponds to the zinc nitrate precursor, and the lower spectrum (green) corresponds to the zinc phosphate microparticles.

The FTIR spectrum of the ZP microparticles is consistent with the characteristic spectrum of zinc phosphate described in other papers. Several authors agree that the vibrations between 800 and 1200 cm^–1^ correspond to the PO_4_^3–^ group.^55,56^ For instance, the spectrum of the obtained ZP microparticles shows a complex of 3 peaks of the stretching PO_4_^3–^ group. Specifically, the bands at 1081 and 1002 cm^–1^ are assigned to the ν_3_ antisymmetric stretching modes of phosphate, and the band at 944 cm^–1^ corresponds to the ν_1_ symmetric stretching mode.^57^ The region at 1598 cm^–1^ is associated with the internal bending vibration of water molecules,^58^ and the band at 3356 cm^–1^ is attributed to the OH stretching.^56^ The band observed at 1353 cm^–1^ could be attributable to residual nitrate ions from the zinc nitrate precursor used during synthesis.^59^ Finally, the bands at 596 and 475 cm^–1^ correspond to the bending mode of PO_4_^3–^.^60–62^

The hydrodynamic diameter of the ZP particles was 4.8 ± 0.5 μm and was close to neutral charges (−1.91 ± 0.18 mV). The TEM micrographs and X-ray diffractogram of the synthesized ZP microparticles are presented in Figure 3A,B, respectively. Regarding the morphology (Figure 3A), microparticles with an elongated shape and different sizes (ranging from about 200 nm to 2 μm) characterized by significant aggregation (typical of precipitated powders) were obtained together with bigger (larger than 5 μm) rectangular particles. Such findings are compatible with the high hydrodynamic diameter determined by DLS measurements. Moreover, due to the local high temperatures generated by the electron beam focusing during TEM analysis, loss of the water component is quite likely, giving rise to the formation of low-contrast areas (with bubble-like structure) surrounding some ZP particles. As for the atomic structure (Figure 3B), the synthesized particles are composed of zinc phosphate tetrahydrate (Zn_3_(PO_4_)_2_·4H_2_O) with a crystal structure of α-hopeite (orthorhombic crystal system).^63^ Such a finding was expected considering the stability of this phase and the mild temperature set for the drying process, below 130 °C, which is the initial dehydration temperature for α-hopeite.^64^ The average crystallite size (*D*) constituting the ZP particles was 35.6 ± 1.6 nm, as calculated from the diffraction peaks corresponding to the (020), (040), and (311) planes with relative intensities of 100%, 91.77%, and 80.58%, respectively.

**Figure 3:**
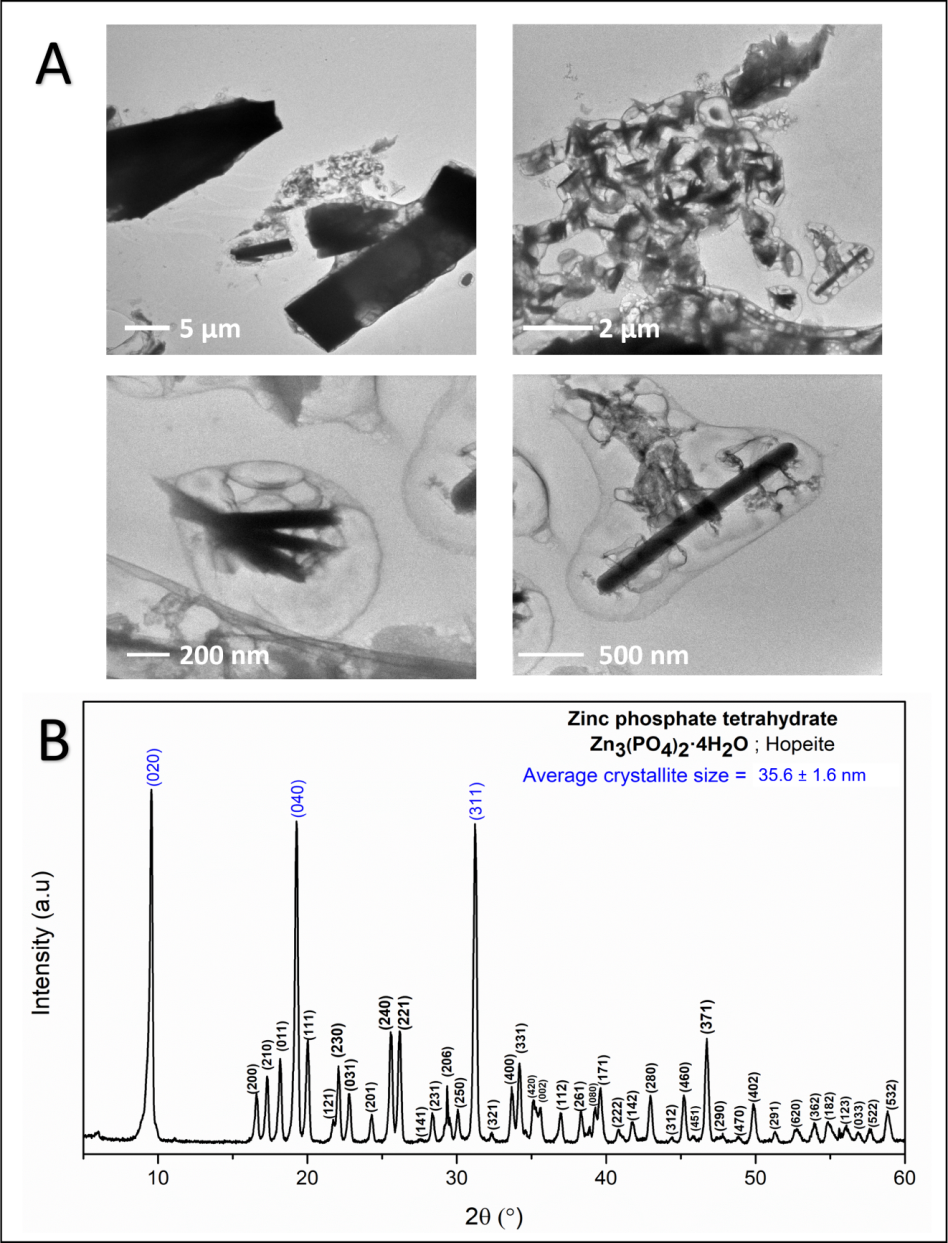
Zinc phosphate micrographs and crystalline structure. (A) ZP particles obtained by chemical precipitation exhibit diverse structures, shapes, morphologies, and sizes. (B) X-ray diffraction pattern of ZP particles synthesized via chemical precipitation; diffraction peaks used to calculate the average crystallite size (D) are marked in blue.

XPS characterization provided the chemical surface composition of ZP microparticles (Table 1), and all of the elements are associated with the synthetic procedure. The high carbon content is not surprising since the drying process and the subsequent sample storage were performed in air. We chose not to remove adventitious carbon by ion sputtering, before XPS analysis, to prevent changes in the inorganic particles’ composition.^65^

**Table 1:** Surface Chemical Composition of ZP Microparticles Expressed as Atomic Percentages; Errors are Taken for Three Replicates

P%	Zn%	Na%	C%	O%	N%
6.1 ± 0.2	9.9 ± 0.1	0.5 ± 0.2	45.5 ± 0.3	36.8 ± 0.2	1.2 ± 0.2

In particular, the Zn/P ratio as calculated by XPS is equal to 1.6 ± 0.2, in good agreement with the theoretical value for zinc orthophosphate (1.5). Typical spectra of Zn 2p_3/2_, ZnL_3_M_4,5_M_4,5_, P 2p, and N 1s are reported in Figure 4. Zn 2p_3/2_, the principal component of the Zn 2p photoelectronic signal, falls at BE = 1022.5 ± 0.1 eV (Figure 4A). The ZnL_3_M_4,5_M_4,5_ Auger signal can be fitted by two components (Figure 4B): the main one, falling at kinetic energy KE = 986.7 ± 0.1 eV, is associated with the nearly degenerated ^1^G, ^3^P, and ^1^D levels, and the secondary one falls at higher KE and can be ascribed to the ^3^F level.^66^ The Auger signal is essential for zinc speciation, since Zn 2p_3/2_ alone is not informative for discriminating valence states for this element.^67^ The sum of BE­(Zn 2p_3/2_) and KE­(ZnL_3_M_4,5_M_4,5_) gives the modified Auger parameter α’ = 2009.2 ± 0.2 eV, in agreement with the formation of zinc orthophosphate.^68^ P 2p_3/2_, the main component of the P 2p doublet, is located at BE = 133.7 ± 0.1 eV (Figure 4C), compatible with phosphate groups. N 1s is detected at about 408 eV (Figure 4D), and therefore, it is ascribed to nitrate ions from the zinc precursor.

**Figure 4:**
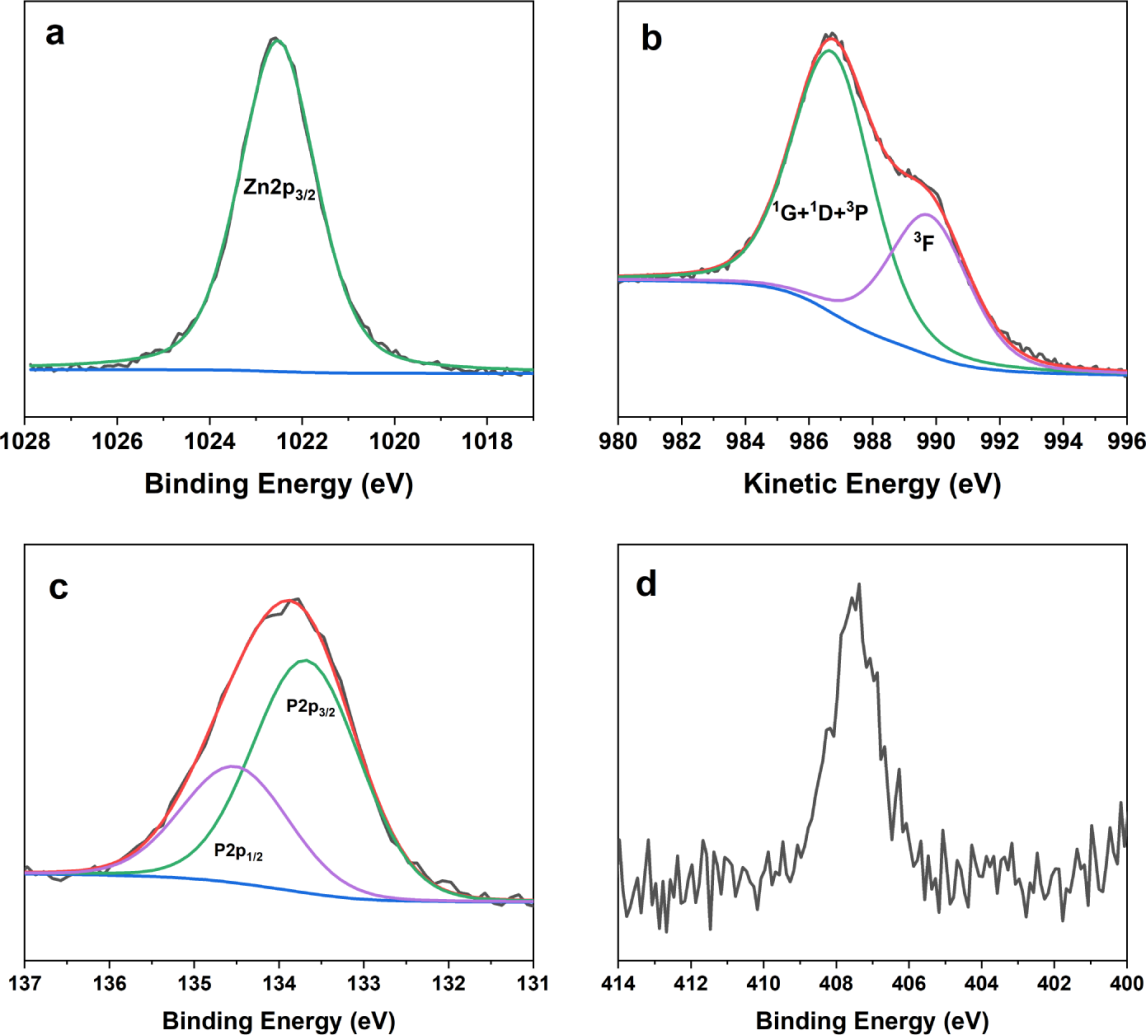
Zn 2p_3/2_ (a), ZnL_3_M_4,5_M_4,5_ (b), P 2p (c), and N 1s (d) regions relevant to ZP powders.

In this study, elongated morphologies of ZP microparticles were observed with a hydrodynamic diameter of approximately 4.8 μm. In contrast, Horky et al. reported ZP particles measuring 0.45 to 1 μm with irregular shapes.^42^ Cai et al. synthesized ZP particles through wet chemical precipitation, resulting in flower-like structures with two-dimensional lamellae measuring 2–5 μm in diameter.^69^ Another research produced ZP particles with a pseudospherical shape and an average size of about 450 nm.^70^ XRD and XPS characterizations confirm the formation of ZP in the present study.

### 3.2 Antibacterial Evaluation

The antibacterial assessment revealed that the ZP microparticles demonstrated concentration-dependent activity against all eight bacterial species tested (Figure 5). Notably, the microparticles exhibited significant inhibitory effects against both Gram-negative and Gram-positive aerobic nosocomial pathogens.

**Figure 5:**
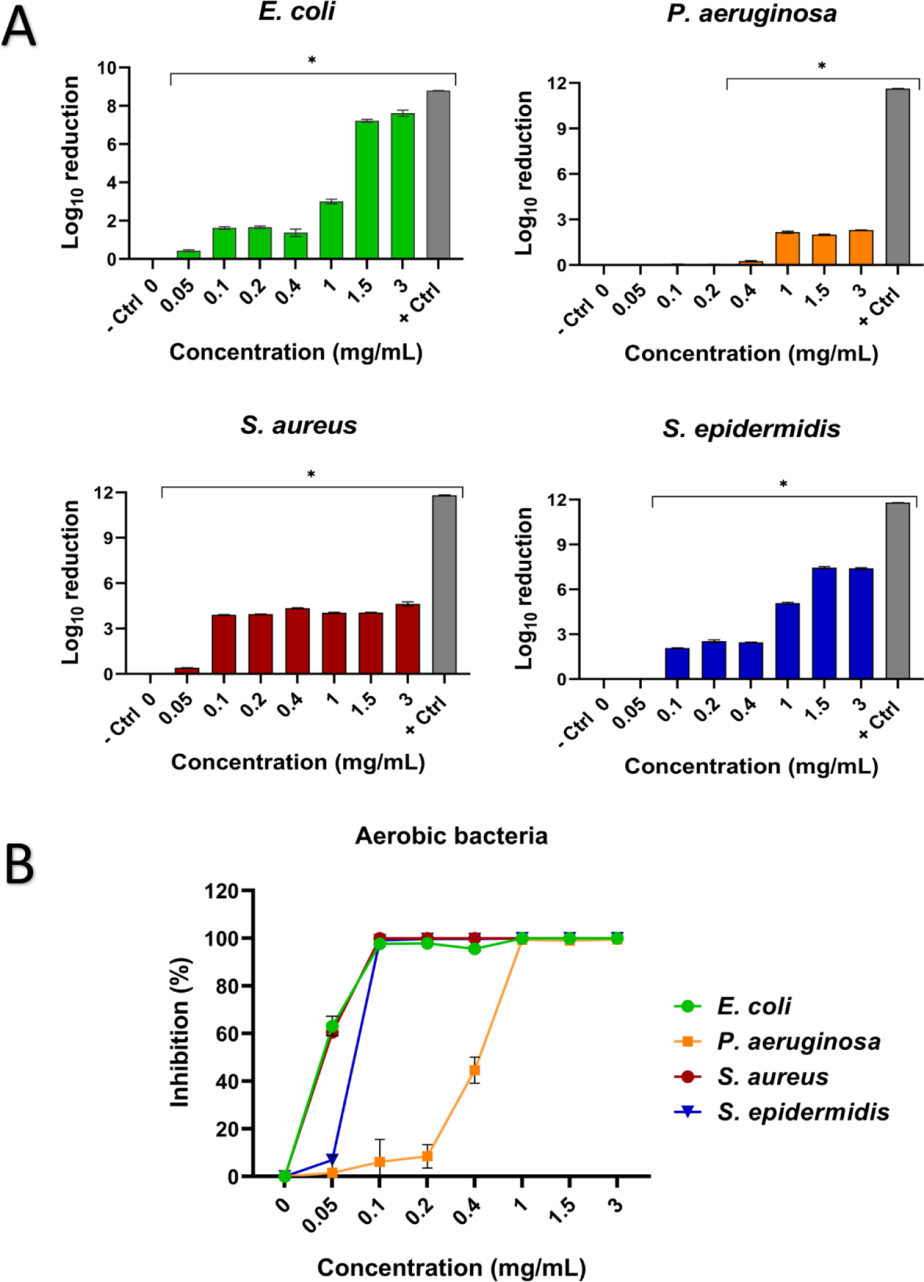
Antibacterial evaluation of ZP microparticles against nosocomial (aerobic) bacteria. (A) Logarithmic reduction of bacterial growth expressed as log_10_ reduction using eq 3. (B) Inhibition percentage of bacterial growth using eq 4. *, *p* < 0.05 versus negative control (bacteria culture without ZP particles; 0 mg/mL concentration). + Ctrl (positive control; bacteria culture with TSB and chlorhexidine 0.2%).

For the Gram-negative bacteria, E. coli exhibited a significant reduction in growth, even at the lowest concentration tested (0.05 mg/mL), and ZP microparticles achieved a 63.2% inhibition of bacterial growth. At higher concentrations (1.5 and 3 mg/mL), the bacterial load was reduced by more than 6 log_10_ units, corresponding to nearly complete inhibition (∼100%). In the case of P. aeruginosa, no significant inhibition was observed at low concentrations of ZP microparticles (0.05, 0.1, and 0.2 mg/mL). However, at 0.4 mg/mL, bacterial growth was significantly inhibited by 44.6%. At the concentrations of 1, 1.5, or 3 mg/mL, the bacterial load was reduced by more than 2log_10_, corresponding to more than 90% of inhibition.

Regarding Gram-positive strains, S. aureus showed significant inhibition at the lowest concentration of ZP microparticles tested (0.05 mg/mL), with an inhibition percentage of 60.5%. At concentrations ranging from 0.1 to 3 mg/mL, the bacterial load was reduced by more than 3log_10_, corresponding to nearly complete inhibition (∼100%). In S. epidermidis, a 0.05 mg/mL concentration did not reduce bacterial growth. However, concentrations from 0.1 mg/mL to the highest tested concentration of 3 mg/mL effectively reduced bacterial growth by more than 90%.

Even though the positive control (0.2% chlorhexidine) achieved the highest logarithmic reduction in aerobic bacterial growth and 100% inhibition in all cases, various concentrations of ZP microparticles, particularly the higher concentrations evaluated, showed significant inhibition of bacterial growth, >90%.

Similar to the case of nosocomial and aerobic bacteria, the oral infection-associated anaerobic bacteria tested were also effectively inhibited in the different concentrations of ZP microparticles, as shown in Figure 6.

**Figure 6:**
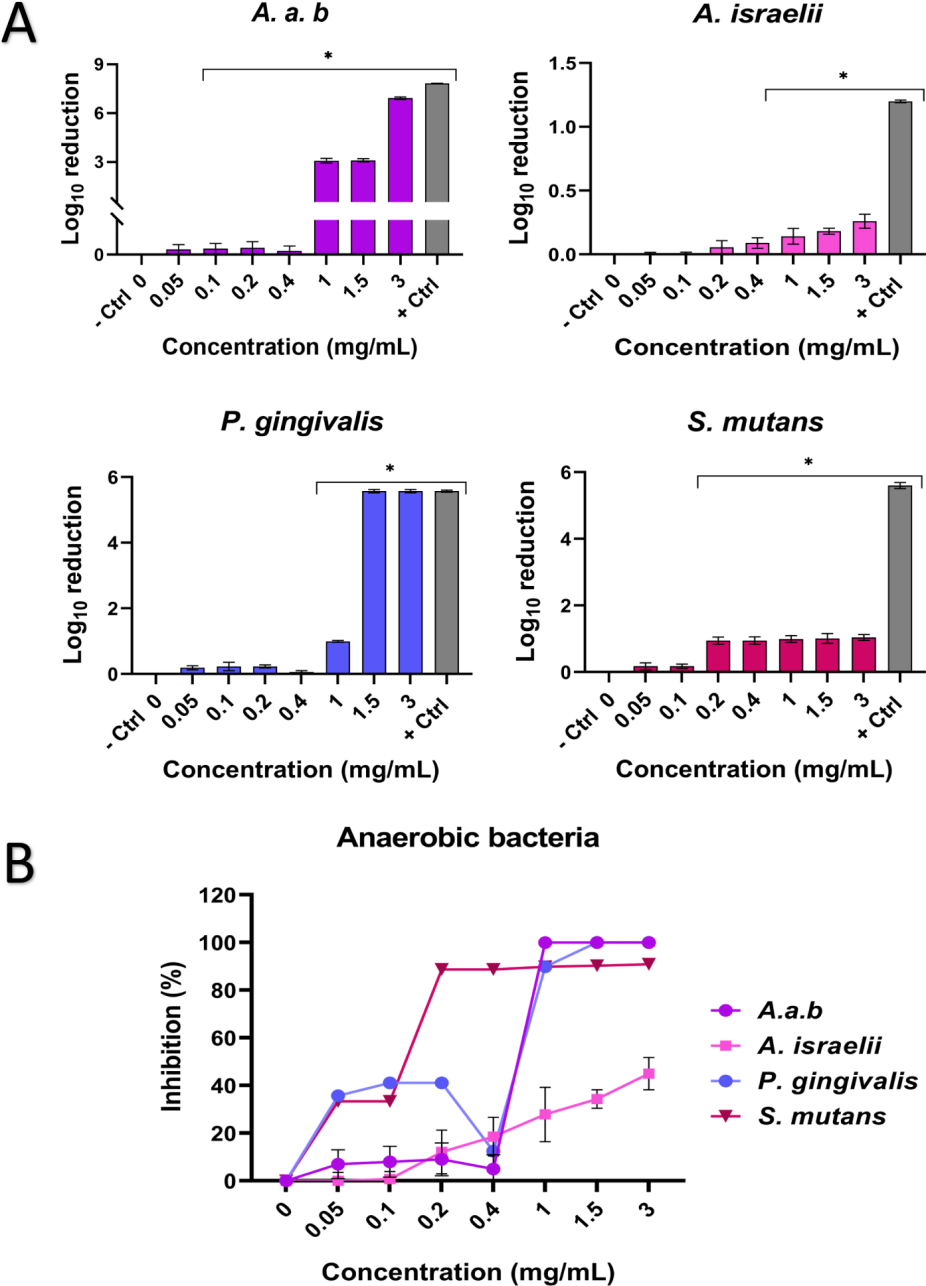
Antibacterial evaluation of ZP microparticles against oral (anaerobic) bacteria. (A) Logarithmic reduction of bacterial growth expressed as log_10_ reduction using eq 3. (B) Inhibition percentage of bacterial growth using eq 4. *, *p* < 0.05 versus negative control (bacteria culture without ZP particles; 0 mg/mL). + Ctrl (positive control; bacteria culture with *Mycoplasma* broth and chlorhexidine 0.2%).

For the Gram-negative bacteria *A. a.* *b*, a significant inhibition was observed from concentrations ranging from 0.1 to 3 mg/mL. At the highest concentration (3 mg/mL), bacterial growth was reduced by more than 6log_10_, corresponding to an inhibition of over 99%. Regarding the other Gram-negative anaerobic bacteria studied, P. gingivalis, the resulting inhibition at concentrations higher than 1 mg/mL was larger than 1log_10_ reduction, corresponding to an inhibition of over 89%. Additionally, this strain was the only in which complete inhibition (100%) was achieved, similar to that of the positive control (0.2% chlorhexidine).

For the anaerobic Gram-positive species (A. israelii and S. mutans), significant inhibition was observed at concentrations of 0.2 and 0.4 mg/mL, respectively. In the case of A. israelii, the highest concentration of ZP microparticles tested resulted in a 0.25log_10_ reduction, corresponding to 45% inhibition. Although this reduction was lower than that of the positive control (0.2% chlorhexidine), which achieved a 1.2 log_10_ reduction, the inhibition observed with ZP microparticles was still considered significant.

Finally, for S. mutans, concentrations ranging from 0.2 to 3 mg/mL resulted in a bacterial reduction close to 1 log_10_, corresponding to an inhibition between 88.7% and 90.9%.

The Gram-negative strains (*A. a. b*. and P. gingivalis) were the most sensitive among the anaerobic species tested. In general, the antimicrobial inhibition of all bacteria tested was proportional to the ZP microparticle concentration; the higher the concentration, the more inhibitory effect was observed. In addition, representative images of the reduction of bacterial growth on agar plates are shown in Figure S1.

Regarding the antibacterial properties, this study found that ZP microparticles exhibited significant antibacterial effects against the eight bacterial strains associated with nosocomial and oral infections. In most cases, the antibacterial potential of the ZP microparticles reached a near 100% inhibition in a concentration-dependent manner, and it was particularly significant at 0.4 and 1 mg/mL. Moreover, the high antibacterial effect observed aligns with and supports findings in other studies that have been investigating the antimicrobial properties of zinc phosphate in nano- and microparticles or coating materials. These results align with findings from other studies evaluating the antibacterial properties of ZP nano- and microparticles (Table 2).

**Table 2:** Summary of Antibacterial Zinc Phosphate (ZP) Material Studies, Describing Their Synthesis Methods, Morphology and Size, Tested Microorganisms, and the Attributed Antibacterial Mechanisms

ZP material	Synthesis method	Morphology and size	Microorganism tested	Attributed antibacterial mechanism	ref.
Zinc phosphate nanoparticles (ZnP-NPs)	Precipitation and biological method in the presence of Enterobacter aerogenes	Average size of 30–35 nm	E. coli, S. aureus and S. mutans.	The nanoparticles’ surface properties and size contribute to their antibacterial effects.	^71^
Coatings of ZnO or Zn_3_(PO_4_)_2_ particles on Titanium substrate	Plasma electrolytic oxidation (PEO)	Porous and rough morphology. With Zn_3_(PO_4_)_2_): 9.85–13.80 μm and with ZnO and Zn_3_(PO_4_)_2_): 3.99–6.57 μm	S. aureus	Bacteriostatic properties. Release of Zn^2+^ from the modified surfaces. Zn^2+^ disrupts bacterial cell membranes, interfere with enzyme function, and generate reactive oxygen species (ROS)	^72^
Zinc phosphate particles	Wet chemical method temperature assistance	Spherical shapes, sizes: 477 and 521 nm. Irregular shape, 452 and 1035 nm	S. aureus,E. coli, Methicillin-resistantStaphylococcus aureus*(MRSA)*	Release of Zn^2+^, interaction with cell wall (more effective in Gram-positive bacteria), ROS, leading to oxidative stress, damaging proteins, DNA, and membranes, and microbiome disruption	^42^
Zinc phosphate particles	Wet chemical method temperature assistance	Irregular shape, size: 452 and 1035 nm	E. coli clinical isolates	Suppressing expression of virulence factors of the fimbrial gene, such as *fimA* in E. coli clinical isolate, which are key for bacterial adhesion and colonization. Enhancing the host’s antioxidant defenses (increased glutathione peroxidase activity)	^44^
Zinc phosphate nanosheets (ZnP-nanosheets)	Biosynthesis using extracellular secretions Aspergillus fumigatus.	2D sheet-like structure 100–200 nm	E. coli, P. aeruginosa, S. aureus, Bacillus subtilis	Release of Zn^2+^ to interfere with bacterial metabolism and function. Disruption of bacterial cell membranes, cell lysis.	^43^
Commercial polymeric membranes of of zinc phosphate	Precipitation and microwave method	-	A. actinomycetemcomitans	Release of zinc ions inhibits bacterial metabolism, disrupts bacterial cell membranes, and inhibits bacterial growth	^39^

El-Sharkawy et al. reported a minimum inhibitory concentration (MIC) of 25 μg/mL for S. aureus, P. aeruginosa, and E. coli, while Bacillus subtilis presented a MIC of 12.5 μg/mL.^43^ Another investigation found that adding ZnO or ZP particles to titanium alloy (Ti-15Mo) substrates enhanced the bacteriostatic effect against species such as S. aureus and S. epidermidis, compared to surfaces without zinc-based particles.^72^

Further research revealed that ZP particles exhibited a high inhibitory effect against S. aureus *in vitro* (IC_50_ ranged from 0.5 to 1.6 mmol/L) and E. coli (IC_50_ 0.8–1.5 mmol/L). However, methicillin-resistant S. aureus (MRSA) was the least sensitive strain (IC_50_ = 1.2–4.7 mmol/L). *In vivo* studies incorporating ZP into the diets of rats or piglets showed a significant decrease in total aerobic and coliform bacterial populations in their feces.^42,44^

It is important to note that few studies have evaluated ZP particles against oral bacteria. Some of these studies have incorporated ZP particles into commercial membranes for dental applications, such as guided bone regeneration, to grant them antibacterial properties. Results indicated a significant reduction in the CFUs against Aggregatibacter actinomycetemcomitans (formerly Actinobacillus actinomycetemcomitans) compared to membranes without ZP particles.^39^ Another example includes the development of zinc oxide-doped phosphate-based glasses, which demonstrated a 1.2 to 1.7 log_10_ reduction of S. mutans in just 2 h.^73^

The exact mechanism by which zinc phosphate (ZP) exhibits antibacterial properties is not fully understood; however, zinc phosphate materials might operate through multiple mechanisms (Figure 7). Some of these mechanisms are related to the capacity for releasing Zn^2+^ ions, which can penetrate bacterial membranes and interfere with enzymatic activity, and generating reactive oxygen species (ROS), which can cause oxidative stress and damage bacterial deoxyribonucleic acid (DNA) and proteins.^39,42–44,73^ Other potential mechanisms involve the interaction between bacterial cells and the morphology of the nano- or microparticles; several studies have suggested that shape and size are essential in their antibacterial activity.^29,74,75^ In the present work, elongated shapes, such as sheets or flakes, can be attributed to the mechanical disruption of bacterial membranes. Also, this interaction increases the negative surface charge on bacterial membranes due to the adsorption of phosphate groups, which enhances the electrostatic attraction between the particles and the microbial surface, facilitating particles’ adhesion and membrane disruption.^31,43^

**Figure 7:**
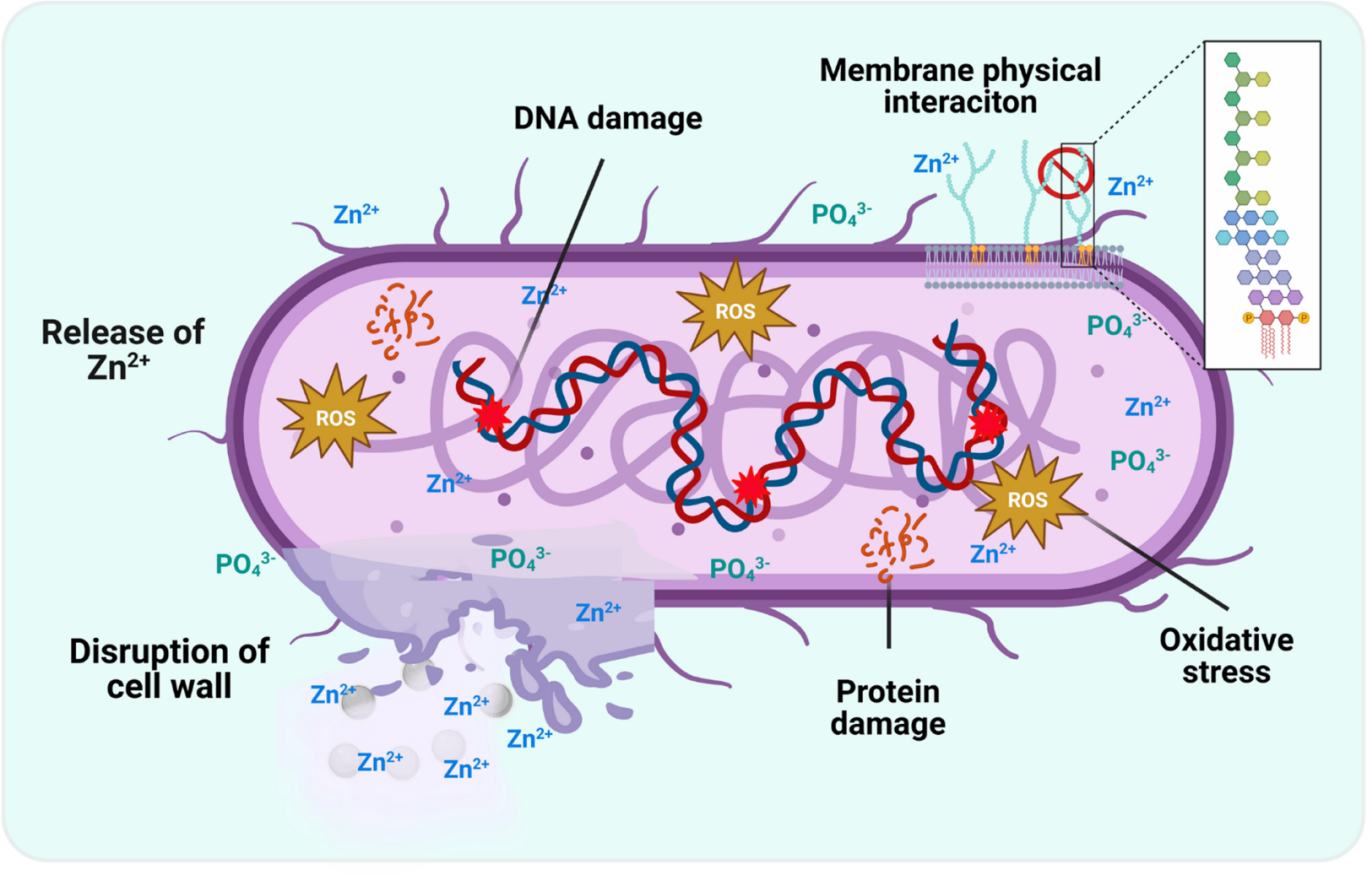
Proposed antibacterial mechanisms attributed to ZP microparticles. Possible interactions such as release of Zn^2+^ ions, generation of ROS, oxidative stress, DNA and protein damage, electrostatic interactions with bacterial membranes facilitated by phosphate groups, and disruption of cell walls. Created in BioRender. Reyes-Carmona, L. (2025) https://BioRender.com/f2p0y6k.

Regarding the differences between Gram-positive and Gram-negative bacteria, in this study, we did not observe a significant difference against ZP particles; both Gram groups exhibited relevant growth inhibition. In contrast with other investigations, where Gram-negative bacteria were more resistant due to their complex outer membrane and efficient efflux systems, Gram-positive bacteria have a thicker peptidoglycan layer but lack an outer membrane, making them potentially more susceptible to Zn^2+^ mediated toxicity and membrane disruption.^76^ The comparable susceptibility observed in both bacterial groups in the present study suggests that the main antibacterial mechanisms of ZP particles, such as ion release and direct interaction with bacterial membranes, are effective across different bacterial cell wall structures.^39,43,77^ This broad-spectrum activity underscores the potential of ZP particles as versatile antimicrobial agents.

In terms of oxygen conditions, both aerobic and anaerobic bacteria showed significant inhibition (>80%) in most cases. However, the literature suggests that the metabolic characteristics of bacteria play an important role in their response to nano- and microparticles. Aerobic bacteria, dependent on oxygen for their metabolism, might be more susceptible to metal ions and oxidative damage induced by ROS generation. In contrast, anaerobic bacteria, which do not require oxygen for growth, might be less vulnerable to these effects.^78,79^

### 3.3 Cytotoxicity Evaluation

The viability percentages of human dermal fibroblasts (HDFa) and human osteoblasts (hFOB) exposed for 24 h to different concentrations of ZP microparticles (ranging from 0.05 to 3 mg/mL) are shown in Figure 8. In general, particle concentrations between 0.05 and 0.4 mg/mL were noncytotoxic to both cell types, as cell viability remained above 80% relative to the positive control. According to ISO 10993-5, a cell viability percentage of less than 70%, compared to the control, can be considered potentially cytotoxic. The viability percentage of fibroblasts and osteoblasts exposed to ZP microparticle concentrations of 0.05, 0.1, 0.2, and 0.4 mg/mL was larger than 70%, in comparison to the control (100%), indicating no cytotoxic effects. Nevertheless, cell viability in percentage was significantly different to the control in the case of fibroblasts, while in the case of osteoblasts, the viability percentage demonstrated no significant difference to the control.

**Figure 8:**
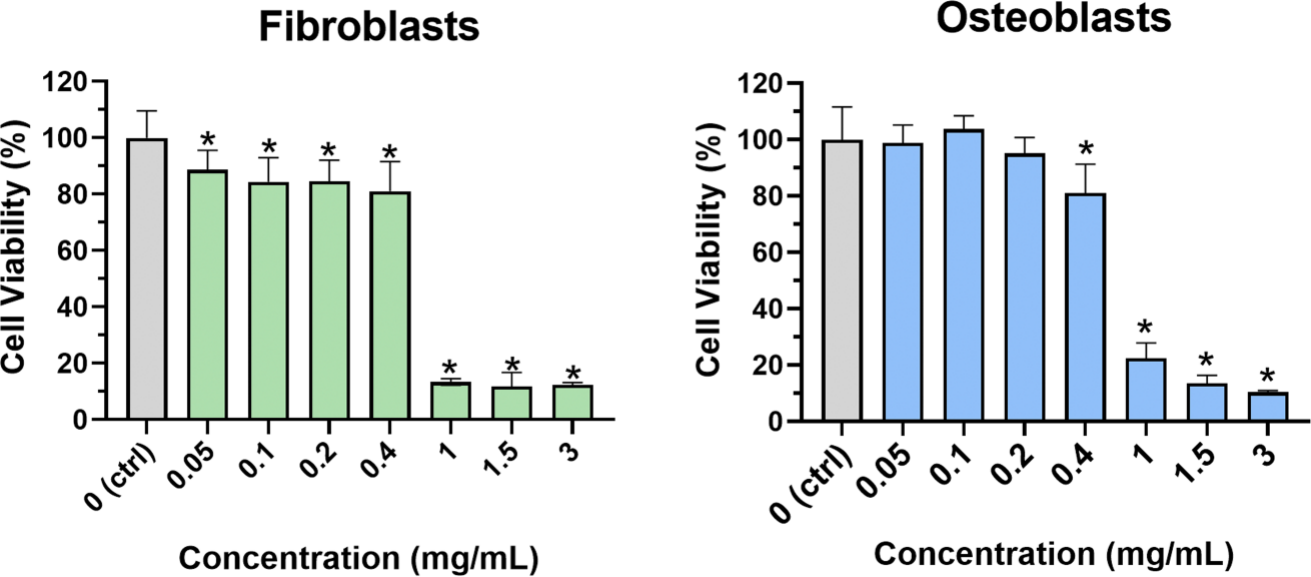
Viability percentage of human dermal fibroblasts (HDFa) and osteoblasts (hFOBs) exposed to different concentrations of ZP microparticles for 24 h, estimated by the MTT assay. *, *p* < 0.05 vs ctrl (cells cultured with culture media without ZP particles).

Regarding the cytotoxic effect, both cell types exhibited similar responses, with a significant decrease in cell viability (below 20%) observed when exposed to 1 mg/mL of higher ZP particle concentration.

Regarding biocompatibility, ZP microparticle concentrations ranging from 0.05 to 0.4 mg/mL were noncytotoxic to both cell types, as cell viability remained above 80% in comparison to the control group. Remarkably, concentrations’ range (0.05 to 0.4 mg/mL) at which ZP microparticles synthesized in the present study were shown to be biocompatible matched the range at which ZP microparticles also demonstrated almost 100% bacterial inhibition for aerobic nosocomial bacteria; E. coli, S. epidermidis, and S. aureus, and anaerobic oral bacteria S. mutants, demostrating the potential of the ZP microparticles to develop safe antimicrobial treatments for different biomedical applications.

Similarly, Leniak-Ziółkowska et al. reported that a Ti-15Mo alloy coated with ZnO or ZP particles positively influenced the viability of MG-63 osteoblast cells, enhancing their cytocompatibility. They noted that zinc promotes both proliferation and viability in osteoblast cells.^72^ In line with these findings, Horky et al. conducted an *in vivo* study and demonstrated that dietary exposure to zinc phosphate NPs did not result in significant alterations in clinical, hematological, or biochemical parameters, indicating a favorable biocompatibility profile and low systemic toxicity.^42^ These consistent results from both *in vitro* and *in vivo* models reinforce the potential of ZP materials for safe biomedical applications.

## 4 Conclusions

Zinc phosphate microparticles, synthesized by using the wet chemical precipitation method, exhibit antibacterial properties against both hospital-acquired infections and oral pathogens while being biocompatible. These findings significantly advance our understanding of this material and provide valuable insights, especially considering the limited research on zinc phosphate as an antimicrobial agent for medical and dental infections. Additionally, the micrometric size and advantageous properties demonstrated in this study suggest that zinc phosphate microparticles could improve safety in various clinical applications. This research opens the door for further exploration and innovation in the use of zinc phosphate in clinical applications and infection control.

## Supplementary Material



## Notes

Representative images of logarithmic reduction of the bacterial growth on agar plates of a Gram-positive nosocomial bacterium (S. epidermidis) and a Gram-negative oral bacterium (P. gingivalis) when exposed to different concentrations of ZP microparticles and their controls (PDF)
