# Supplementary material for: Zinc Phosphate Microparticles against Nosocomial and Oral Bacteria: Synthesis, Analytical Characterization, and Biocompatibility
Source: ACS Omega. 2025 Jun 12;10(24):25829–41. doi: 10.1021/acsomega.5c02071 (PMC12199044; doi:10.1021/acsomega.5c02071)
Supplement: Supplementary file 1 [file ao5c02071_si_001.pdf]

Supplementary Material

Zinc phosphate microparticles against nosocomial and oral bacteria: synthesis, analytical characterization and biocompatibility

Authors: Lorena Reyes-Carmona[a,1,*], Margherita Izzi[b,1], Rosaria Anna Picca[b], Maria Chiara Sportelli[b], Gina Prado-Prone[a], Phaedra Silva-Bermudez[c], Sandra E. Rodil[d] , Nicola Cioffi[b,*], Argelia Almaguer-Flores[a,*].

Affiliations: [a] Laboratorio de Biointerfases, División de Estudios de Posgrado e Investigación, Facultad de Odontología, Universidad Nacional Autónoma de México. Circuito exterior s/n, Ciudad Universitaria, CDMX, 04510, México.

[b] Dipartimento di Chimica, Università degli Studi di Bari Aldo Moro. Via E. Orabona 4, 70125 Bari, Italia.

[c] Unidad de Ingeniería de Tejidos, Terapia Celular y Medicina Regenerativa; Instituto Nacional de Rehabilitación Luis Guillermo Ibarra Ibarra, Av. México-Xochimilco No. 289 Col. Arenal de Guadalupe, C.P. 14389, CDMX, México.

[d] Instituto de Investigaciones en Materiales, Universidad Nacional Autónoma de México. Circuito exterior s/n, Ciudad Universitaria, CDMX, 04510, México.

[1] These authors contributed equally to the work as first authors.

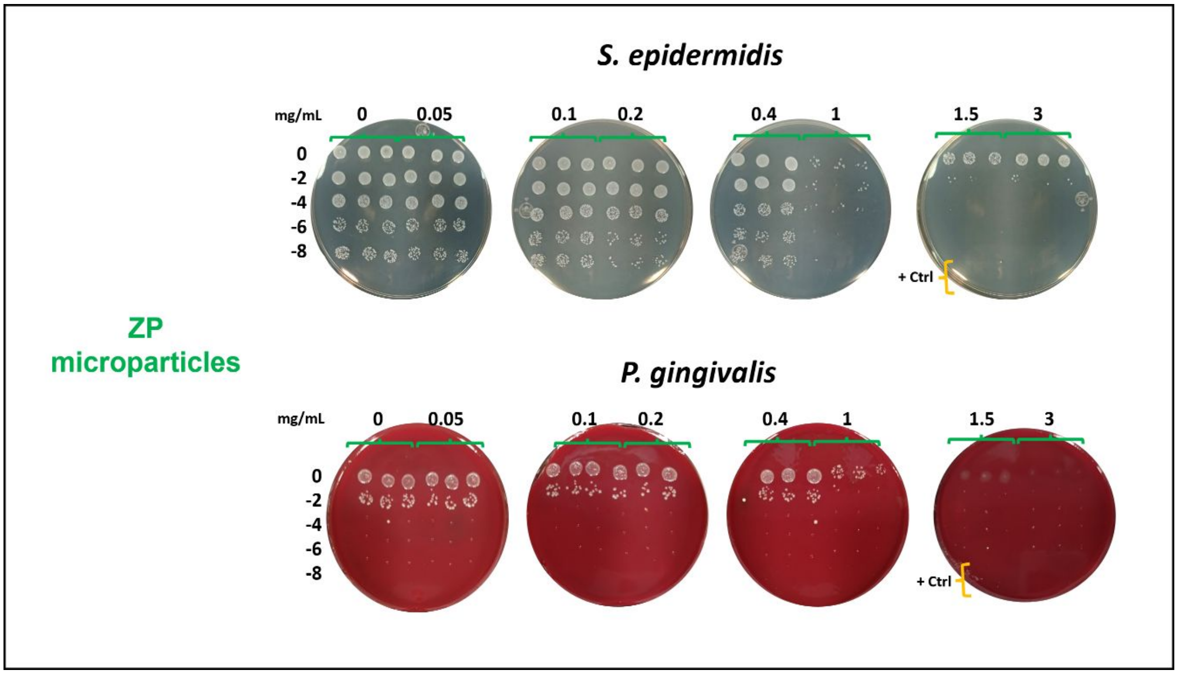

Figure S1. Representative images of logarithmic reduction of the bacterial growth on agar plates of a Gram-positive nosocomial aerobic bacteria (*S. epidermidis*) and a Gram-negative oral anaerobic bacteria (*P. gingivalis*) when exposed to different concentrations of ZP microparticles and their controls.
